# A Survey of Dairy Cattle Behavior in Different Barns in Northern Italy

**DOI:** 10.3390/ani10040713

**Published:** 2020-04-19

**Authors:** Daniela Lovarelli, Alberto Finzi, Gabriele Mattachini, Elisabetta Riva

**Affiliations:** 1 Department of Environmental Science and Policy, Università degli Studi di Milano, 20133 Milano, Italy; 2 Department of Agricultural and Environmental Sciences, Università degli Studi di Milano, 20133 Milano, Italy; alberto.finzi@unimi.it (A.F.); mxlelex@libero.it (G.M.); elisabetta.riva@unimi.it (E.R.)

**Keywords:** cow behavior, environment, heat stress, lying time, monitoring, sensors, Precision Livestock Farming, THI

## Abstract

**Simple Summary:**

The climate crisis is accompanied by an increasing number of heat waves that negatively affect the behavior of dairy cows and their welfare. To understand if and how this is affecting farms in Northern Italy, a survey was carried out on eight cattle farms located in the Lombardy region. Three periods were monitored for one year (thermoneutral, hot and cold seasons) using environmental sensors installed in the barn and accelerometers mounted on the hind leg of groups of cows. From the results, it emerged that cows react to high air temperature and humidity conditions by reducing their lying time, which negatively affects milk production. Four out of the eight investigated farms showed that the negative effects caused by heat stress were evident. Hence, the farmer should consider the possibility of improving the barn structure, for example with an efficacious forced ventilation system. Cattle welfare is the first step towards healthy and productive cows.

**Abstract:**

Due to its increasing pressure on dairy cows, studies that investigate how to cope with heat stress are needed. The heat stress affects multiple aspects of cows’ lives, among which their behavior and welfare. In this study, a survey was carried out in eight farms located in Northern Italy to monitor and evaluate the environmental aspects of the barns and the behavioral responses of dairy cows. For one year, three periods were monitored: thermoneutral (T_S), hot (H_S) and cold (C_S) seasons. Temperature and relative humidity were measured by environmental sensors, and lying vs. standing time, number of lying bouts and their average duration were collected by accelerometers. The temperature-humidity index (THI) was quantified inside and outside of the barn. Results show that at the increase of the THI, behavioral adaptations occurred in all the farms, especially with a reduction of lying time and an increase of respiration rate. Four of the eight farms need interventions for improving the cows’ welfare. Here, environmental problems should be solved by introducing or improving the efficacy of the forced ventilation or by modifying the barn structure. Monitoring dairy barns with sensors and Precision Livestock Farming techniques can be helpful for future livestock farming to alert farmers on the need for their interventions to respond immediately to unwanted barn living conditions.

## 1. Introduction

In recent years, the behavior, health and welfare of reared animals, and specifically of dairy cattle, have been increasingly put under pressure by extreme heat wave events. If not managed with proper cooling solutions, the hot periods, which hit during summer in temperate climates, negatively affect the living conditions of dairy cows. It is widely known that cows suffer from high temperature conditions and that heat waves negatively affect production and reproduction aspects [1,2,3], especially when these conditions of high air temperature and relative humidity last long for days. In the most extreme conditions, cattle mortality has also been found to increase [1,2,4]. Efficient production (i.e., milk and/or meat) and reproduction (to reduce the unproductive phases) are the main aspects on which farmers focus, mostly because of the direct relation with their economic return. However, production and reproduction are also associated the cows’ welfare and the environmental and social sustainability [5], which must not be disregarded. In particular, the cattle behavior adapts to respond to excessive heat problematics by, first of all, varying the lying or standing time and the feed intake, which affect milk yield and animal health [6].

Therefore, a reduction of the negative effects caused by heat stress on dairy cows plays a fundamental role from the farmer’s point of view, who is inclined to pay for technological solutions that permit the monitoring of the reared animals, the surrounding environment and the barn management [7,8]. The emerging issue of heat stress is affecting most of all the livestock farming systems of temperate climates as a consequence of the current climate crisis. These areas are more susceptible to the emergence of heat stressing conditions [9,10]. In temperate climates where cows are reared commonly in open barns, important interventions have been done already to make the environment more adequate for animals’ living conditions. In particular, building new barns is obviously the optimal option, as it allows the introduction of the best technologies and knowledge about ideal natural and forced ventilation, shades, building materials and building engineering. When this is not possible, supporting solutions must be adopted. Among the main options, forced ventilation is one of the most adapted and unavoidable interventions to be introduced. In addition, shades offer a great protection against solar radiation and heat stressing conditions [11], as does the possibility of introducing cow showers in the barn to lower down the body temperature [12].

A very big share of the Italian livestock farms are located in Northern Italy. According to the Italian institute of statistics (ISTAT) [13], at the end of 2018, about 70% of the Italian dairy and meat cattle and 87% of the Italian swine were reared in this area, while other typologies of animals were reared in lower amounts (about 7% of sheep and 30% of goats, with respect to the national value). Therefore, the wide majority of high-income livestock activities are located in Northern Italy. Here, the composition of livestock farms is still quite variable, since both big and innovative farms as well as small and traditional farms are present. Commonly, the biggest and most innovative farms are located in plain areas, while on the mountains, small farms descriptive of the local traditional activities can be frequently found [14]. The climatic conditions of Northern Italy are also quite different, although this area is subject to important heat waves. Given that it is a basin area surrounded by the mountain range of the Alps, the air exchanges are more limited than in other countries. In fact, air pollution worsens due to the presence of livestock activities [15], which are responsible for a wide share of ammonia, particulate matter and methane emissions [16]. The effect of ammonia as an acidifying substance and precursor of particulate matter formation and the effect of methane as co-participant to global warming have been widely studied [17,18,19]. Solutions to abate such emissions have been investigated and introduced, among which include air scrubbers, covered tanks for slurry storage, anaerobic digestion plants, etc. [20,21,22]. Despite these huge efforts in improving the livestock systems to reduce damages to the environment, the climate crisis weakens these efforts. Because of the susceptibility of animals, and especially of dairy cattle, to warm air temperatures and high humidity, the climatic conditions of Northern Italy are pushing dairy cattle under pressure, and the heat stress reinforces the issue [23]. Farmers, researchers and stakeholders, such as policy makers and private companies, must identify the best way to deal with hot climate and unexpected dangerous heat waves. Since farmers may not be completely aware of the possible interventions to mitigate heat stress, research must be directed towards training and teaching farmers. In particular, technology, wireless connectivity, sensors and monitoring tools are giving the opportunity to farmers to monitor animals and get advantages from this animal-to-animal control [24,25]. This is recognized under the heading of Precision Livestock Farming (PLF), which is a multidisciplinary science that allows us to manage animals by individually adopting a continuous real-time monitoring of health, welfare, production and reproduction aspects and their environmental impact [26,27].

In this context, the aim of this study was to monitor a sample of dairy cattle farms located in Northern Italy in order to: (i) point out the cows’ behavioral response to seasonal environmental conditions, and (ii) evaluate the circumstances in which strategies should be introduced to improve the animal welfare, with the final scope of reducing or even potentially avoiding production and reproduction losses.

## 2. Materials and Methods

During the year 2018–2019, three measuring surveys lasting one week each were completed on eight livestock farms in the Lombardy region, located in Northern Italy. In every farm, a group of cows was monitored. In particular, 10 dairy cows per farm were selected randomly during the first survey and, as close as possible, the same cows were maintained in the subsequent monitoring surveys. Hence, a total of 240 cows were monitored.

The three measuring surveys were performed per season: one in the thermoneutral season (spring or autumn) (T_S), one in the hot season (summer) (H_S) and the last one in the cold season (winter) (C_S). During every survey, in every farm, data were collected using environmental sensors to monitor the farm environmental conditions, and accelerometers to analyze the animals’ behavior. 

With the environmental ones, data about climatic variables inside the barn were collected and analyzed to evaluate the living conditions of dairy cows. In particular, temperature and relative humidity in the barn were used to quantify the temperature-humidity index (THI) and to evaluate the possible presence of unwanted conditions, above all being heat stress. Heat stress is defined as a condition in which cows suffer from excessive temperature and humidity in the barn, which is clearly evident at the THI threshold commonly identified at THI ≥ 72 [28], but it starts emerging also at a lower THI. Hence, we consider that cows start suffering from heat stress already at lower THI values. Secondly, accelerometers were positioned on the selected dairy cows (i.e., 10 cows per farm per period) to evaluate their activity and to understand the health and welfare conditions of cows in response to the environmental conditions. Details about these tools are given in the following sections (Section 2.2 and Section 2.3).

The data analysis was carried out using the software SAS 9.4 (TS1M3, 2012, SAS Inst. Inc. Cary, NC, USA). The differences between the seasons (measuring periods) were checked with the GLM procedure.

### 2.1. Livestock Farms

**Figure 1:**
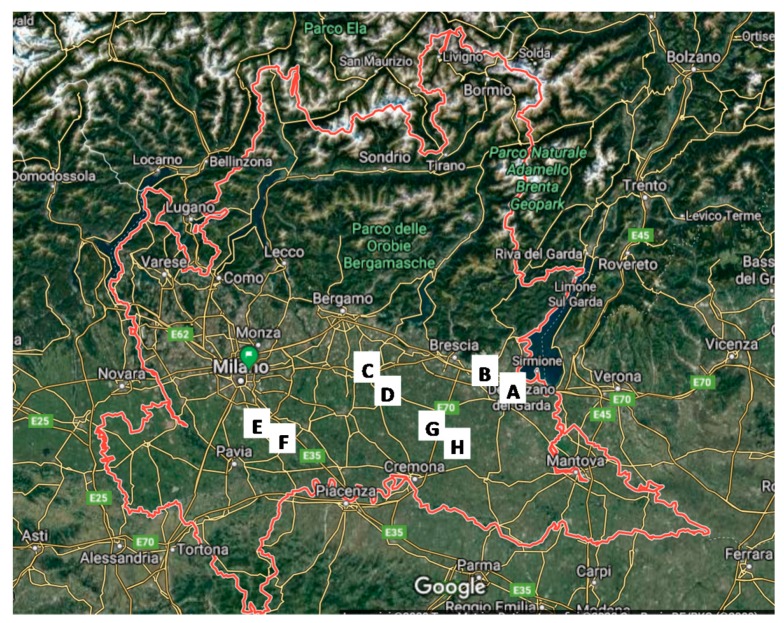
Map of Lombardy region in which the localization of the eight sampled farms is reported. Farms A and B are in Brescia province; farms C, D, G and H are in Cremona province; farm E is in Pavia province and F in Lodi province.

The eight sampled farms are hereby named A, B, C, D, E, F, G and H, and are characterized by the following main features. All farms are located in the same region, and are grouped in pairs in terms of distance in order to have a restrained climatic difference between the closest ones and to reduce the effect from uncontrollable external variables. For this reason, the paired farms were monitored during the same week. Figure 1 shows the geographical distribution of the farms in the region. 

All the cows monitored by means of accelerometers were Holstein Friesian. Housing consisted of free-stall pens in a loose-housing layout. The herd management, cubicles, feeding places and bedding materials differed in the different farms, and also the building, ventilation system and lighting specifically differed, as reported in Table 1. None of the farms had an external paddock or an automatic milking system.

The farms are located in four provinces of Lombardy (i.e., Brescia, Cremona, Pavia and Lodi), and they were selected upon two levels: first, the geographical localization, and second, the availability of farmers. The geographical distribution was defined in order to be representative of the climatic conditions of the plain area of Lombardy (namely the Po Valley), which is where most livestock farms are present. The second selection was completed according to the availability of the farmers to be included in the project and their ability to be available to have sensors installed in the barn and on a restrained selected number of animals. Among the eight farms, farm E is the experimental one managed by the University of Milan (“Az. Menozzi,” Landriano, Pavia province).

### 2.2. Climatic Variables

HOBO U12 Temp/RH/Light/External Data Logger (Onset Computer Corporation, Bourne, MA, USA) sensors were installed in every farm in two different positions in the barn and at the height of 2 m in accordance with Mattachini et al. (2013) [29]. They were installed inside the barn to collect data about the environmental living conditions of dairy cows. They continuously collected data during one week in every data collection period. Data include air temperature in the barn and relative humidity recorded every 30 min. With these data, THI is calculated. Although there are several different equations for quantifying THI, in this study the equation by ASABE (2006) [30] was selected (Equation (1)):

(1) $$THI=T+0.36\times T_{dp}+41.2$$

where T = dry bulb temperature (°C), T_dp_ = dew point temperature (°C) and THI = Temperature-humidity index.

Additionally, the climatic conditions external to the barn were obtained from the weather stations closest to every farm. The meteorological variables were downloaded from the website of the Regional Agency for the Protection of the Environment (ARPA) [31], and were air temperature (T; °C) and relative humidity (RH; %). T and RH were used to calculate the THI of the external conditions of the barn. THI was calculated also in this case with Equation (1).

### 2.3. Monitoring of Cows’ Behavior

HOBO Pendant G Data Loggers (Onset Computer Corporation, Pocasset, MA, US) were positioned individually on the hind leg of 10 dairy cows of every farm to record continuously their activity and, in particular, to record the leg orientation and detect the lying (or standing) activity. This device recorded data at 1-min intervals for the whole period. Also, this instrument was installed three times a year in each of the eight farms and data were recorded continuously for about one week in every data collection period. The HOBO Pendant sensor was mounted on the leg by means of tape and a plastic tough leg band in order to have the sensor positioned with the x-axis of the data logger perpendicular to the floor. The degree of vertical tilt of the x- and z-axis was used to quantify the behavior of the animal and, in particular, the standing or lying behavior [29]. 

These data were then used to quantify the standing and lying time per day (h/d), the frequency of lying bouts (n bout/d) and the duration of the lying bouts (min/bout) per cow and per day. Moreover, the respiration rate of 10 lying down cows per farm per survey was measured for 1 min at the beginning and at the end of every survey period. This information was used to identify if a relation emerged between the number of breaths and heat stress. 

## 3. Results

### 3.1. Results Per Period

For the eight farms analyzed together, and only split into the three different survey periods (thermoneutral T_S, hot H_S and cold C_S seasons), Table 2 reports the average results of the environmental monitoring carried out with environmental sensors and data collected from the meteorological stations. Then, Table 3 shows the average results of the behavioral monitoring in terms of lying vs. standing time, number of lying bouts and their duration. For all the parameters considered (i.e., THI, lying time and number of lying bouts), the results of the three periods were statistically different.

Although the farms are geographically close to each other, or at least at a reduced distance, some environmental differences can be highlighted among the farms. Standard deviation, minimum and maximum values of every variable can differ, especially when relative humidity is evaluated. Among the three periods, the average temperatures differ, being on average 19.0, 26.3 and 6.3 °C, respectively, for the T_S, H_S and C_S. In all the cases, the average temperature in the barn results are higher than the temperature monitored in the meteorological stations close to the farms, although in this case, no statistically significant difference emerges. On the opposite, the RH external to the barn results are higher than the internal RH of the barn, but no statistically significant differences are found. Generally, for RH, there are bigger differences, but from the monitoring of both sensors in the barn and the external data from the meteorological stations, the cold season results are the one with the highest RH.

Considering THI, in T_S and H_S the value is higher in the barn than outside (63.0 and 60.9 in T_S, respectively, in and out of the barn, and 71.8 and 67.5 in H_S, respectively), while the opposite occurs for the C_S (48.4 and 49.8, respectively, in and out of the barn).

Table 3 reports mean values, standard deviation and minimum and maximum hours per day of lying and standing time of the monitored cows, as well as of the number of lying bouts per day and their duration. These results are achieved from the processing of accelerometer data. A statistical analysis is performed in the Section 3.3. to evaluate the differences among farms. 

From these values, it emerges that the average lying time of the eight farms is affected by seasonality: H_S shows the lowest values of lying time, while C_S shows the highest and is also characterized by the lowest standard deviation. Since standing time is the difference between 24 h/d and the lying time (h/d), standing is identified by the opposite trend of lying time. 

As expected, with hot air temperature and high relative humidity, animals lie down less than during temperate-cold periods that are more adapted to their welfare conditions. This aspect emerges also from the analysis of lying bouts: the number of lying bouts is the highest during T_S and H_S periods and the bouts duration is the shortest (70.0, 65.4 and 81.4 minutes/bout, respectively, for T_S, H_S and C_S). Hence, animals rest more during the cold season. Regarding the bouts’ number and duration, T_S shows the widest variability, which may mean that other aspects could affect this result (e.g., changes in the photoperiod).

### 3.2. Environmental Results Per Farm

**Figure 2:**
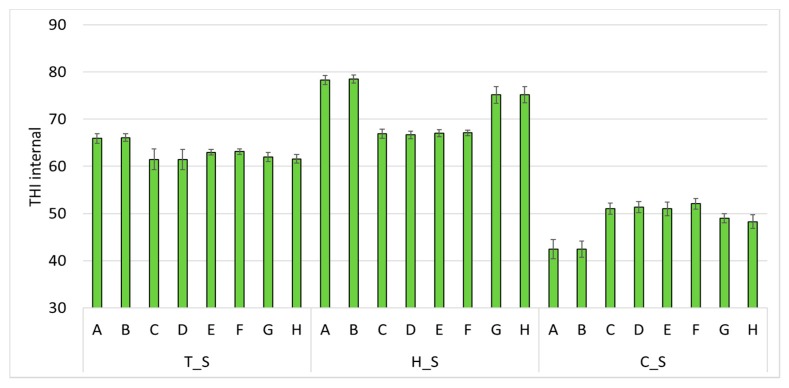
Average daily THI in every farm (A to H) during the three measuring periods (thermoneutral = T_S, hot = H_S and cold = C_S). Vertical bars express the standard deviation.

Considering the results of THI per farm and per survey, Figure 2 shows the THI in the monitored farms. During T_S, quite restrained differences emerge among farms, as the climatic conditions are similar. All the eight farms show the THI included within 61–67, which is not alarming but should not be disregarded. Similarly, during C_S, the values are close to each other for all farms (48–52) except for farms A and B that highlight lower THI (42–43). Seeing that these two farms matched in the geographical localization in the region, specific climatic conditions may be responsible for this result. Finally, H_S shows a bigger variability. In particular, the two farms A and B show the highest average THI per survey, equal to 78–79. Instead, THI levels in farms G and H are 75–76. In the other four farms, THI is always included within the range of 66–68, which is below the threshold of heat stress for dairy cattle (i.e., 72). 

**Figure 3:**
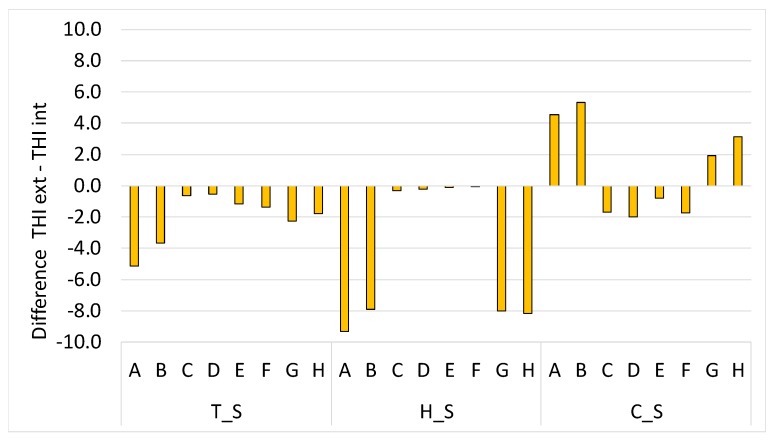
Average difference between the THI outside the barn (THI external) and the one inside the barn (THI internal) for every farm (A to H) and period (thermoneutral = T_S, hot = H_S and cold = C_S) of the monitoring.

From this analysis, it seems that farms A and B as well as farms G and H may have insulation problems due to the barn structure and/or an inefficacious cooling system. For these farms, this is confirmed by the results reported in Figure 3, where the difference between the external THI (quantified with the data from the meteorological station) and the internal THI (measured in the barn with the data logger) is the widest in all the 3 periods. On the opposite, the other four farms (i.e., C, D, E and F) show a THI in the barn slightly and constantly lower than the external THI; therefore, no effect appears from possible bad insulation and/or a bad cooling system. 

Hence, the four farms A, B, G and H, as a result, are badly insulated and/or badly cooled down, which may be responsible for negative effects on the health, behavior and animal welfare. Instead, the other four farms results are sufficiently insulated and/or cooled to respond to hot periods.

### 3.3. Behavioral Results Per Farm

**Figure 4:**
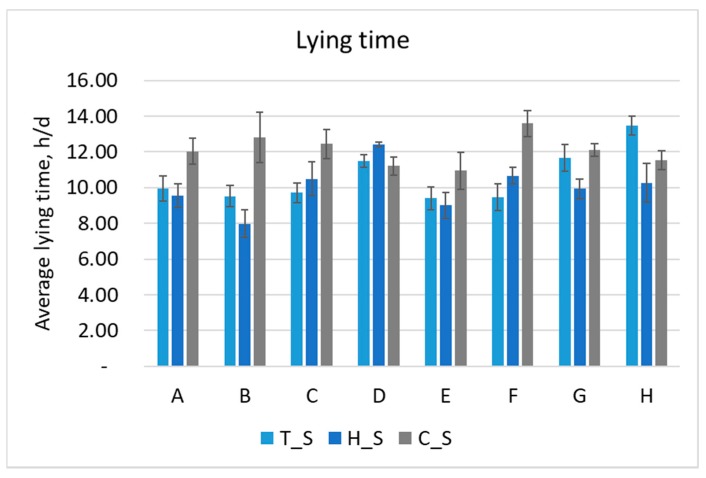
Average lying time in every farm (A to H) for the three measuring periods (thermoneutral = T_S, hot = H_S and cold = C_S). Vertical bars express the standard deviation.

The daily lying time of cows is known to be a very important parameter to evaluate for acceptable animal welfare and production aspects. In the monitored farms, the average lying time is included within the range of 6.5 and 14.4 h/d, with the lowest value (6.5 h/d) representative of H_S and the highest (14.4 h/d) of C_S. Figure 4 reports these results.

During C_S, in which the average THI never exceeds 52, the lying time of the monitored cows ranges between 10.9 (in Farm E) and 13.6 h/d (in Farm F). During the other two periods, instead, a larger variability is reported. In particular, Farm D shows the best results, since restrained differences in the lying time of cows emerge during the three seasons (with a range between 11.2 and 12.4 h/d, in C_S and H_S, respectively). On the contrary, Farm B shows the worst result of lying time during H_S, with the average value of 8.0 h/d of lying, which is the lowest result recorded. In H_S, Farms C, F and H show good values for the lying time (average in the range between 10.3–10.7 h/d). Together with the previously mentioned Farm B, Farms A and E show low values of lying time, and are included in any case between 9.0–9.6 h/d of average lying time.

In T_S, the variability of results among farms is also quite big. In particular, Farm H is the one in which the highest lying time is recorded; in all the measurements, it ranges between 12.8 and 14.0 h/d. Steady values are monitored also in Farm D, with results between 11.1–12.1 h/d of lying, and in Farm G, with a trend very similar to Farm D, except for a maximum value recorded for lying time equal to 13.1 h/d. For this survey as well, Farms A and C ranged between a minimum of 9.1–9.2 h/d and a maximum of 10.6–11.0 h/d, while Farms B, E and F had lying time not exceeding 10.4 h/d and with the lower values close to 8.5 h/d. 

Focusing on the H_S, a statistical analysis was carried out among the farms to identify if the differences in lying time were statistically significant. Table 4 reports these results. In particular, Farm B results in the lowest lying time and Farm D results in the highest with statistical significance (*p* < 0.001). 

**Figure 5:**
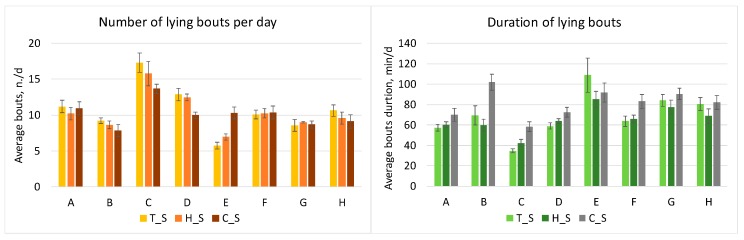
Average number of lying bouts per day (left figure) and duration of lying bouts (right figure) for the eight farms (A to H) in the three measurement periods (thermoneutral = T_S, hot = H_S and cold = C_S). Vertical bars express the standard deviation.

Figure 5 reports the average number of lying bouts per day and their average duration. Although there seems to be a seasonal trend, it seems also that these variables are not strongly related to the THI of every period. Nevertheless, in the cold period, the number of lying bouts is reduced (on average 10.06 ± 1.79 n./d, with respect to 10.70 ± 3.30 n./d and 10.36 ± 2.66 n./d for the thermoneutral and hot periods, respectively) and the duration of the lying bouts is longer (on average 81.4 ± 14.2 min/d, with respect to 70.0 ± 22.1 min /d and 65.4 ± 13.0 min/d for T_S and H_S, respectively).

Farms F and G are the two with the most reduced differences in number of lying bouts along the three periods (10.1–10.4 and 8.6–9.0 n. bouts/d, respectively, in farm F and G), while Farms C and E show the extreme values, respectively, with the highest results in number of lying bouts in Farm C and the lowest in Farm E. The latter also has an opposite seasonal trend respect to the other farms. Regarding the duration of the lying bouts, during the cold period, they generally last much more than in the other periods. Moreover, Farm C, that which the highest number of lying bouts, also has the lowest duration in bouts (34–58 min/bout, respectively, in T_S and C_S), meaning that these cows rest less than in the other farms.

**Figure 6:**
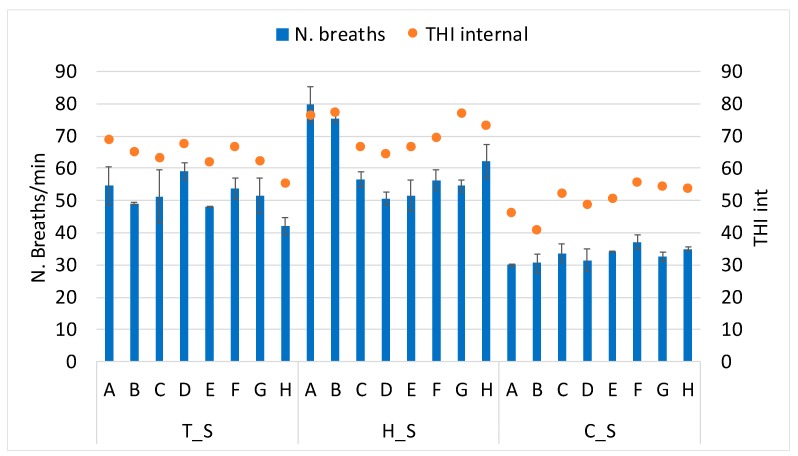
Relation between THI internal in the barn and the respiration rate (number of breaths/min) during the three periods (thermoneutral = T_S, hot = H_S and cold = C_S) in each sampled farm (A to H). Vertical bars express the standard deviation.

As mentioned, the respiration rate (n. breaths/min) was also measured in the random sample of cows during every survey. Figure 6 shows the average number of breaths measured per minute in the eight farms during the three annual surveys. In the figure, breaths are related to the internal THI in the barn, so that the direct proportionality between breaths and THI emerges. In the four farms characterized by adequate insulation and/or cooling (i.e., C, D, E and F), breaths are not deeply affected by the period of the survey. Instead, the four farms characterized by bad insulation and/or inadequate cooling system have the worst seasonal variability with respect to the average number of breaths, which once more can affect negatively the cows’ welfare during the summer. 

### 3.4. Relations Between Environmental and Behavioral Aspects

**Figure 7:**
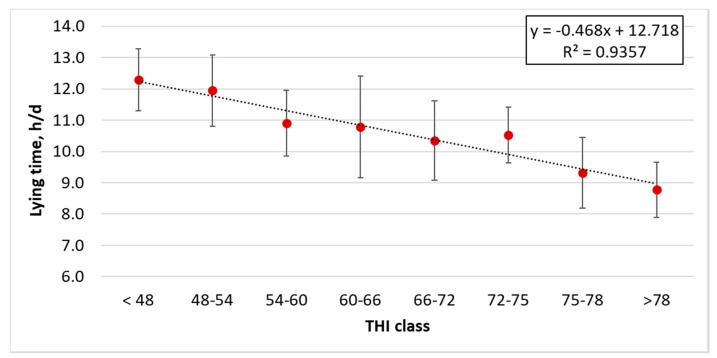
Relation between the lying behavior (lying time; h/d) of the dairy cows monitored at different classes of THI. Vertical bars express the standard deviations.

Environmental conditions and cow behavioral responses were analyzed together to identify their possible relations. Even if there is a large variability in the results that is mostly caused by the seasonality and the specific cattle and farms characteristics, Figure 7 shows the decrease in lying time at the increase in THI. This analysis results from all the cows monitored in the eight farms. Following Brown-Brandl et al. (2005) and Provolo and Riva (2008) [1,28], the THI was clustered in eight classes as follows: (a) THI < 48, (b) 48 ≤ THI < 54, (c) 54 ≤ THI < 60, (d) 60 ≤ THI < 66, (e) 66 ≤ THI < 72, (f) 72 ≤ THI < 75, (g) 75 ≤ THI < 78 and (h) THI ≥ 78.

The 84% of data reported in Figure 7 lie in the classes below the THI threshold of 72, over which the condition is recognized as heat stressing. However, almost 50% of these data lie in the two classes slightly below this threshold (i.e., classes 60–66 and 66–72), meaning that half of the cows are already close to suffering. A clear trend results in the expected reduction in lying time when the THI is high.

**Figure 8:**
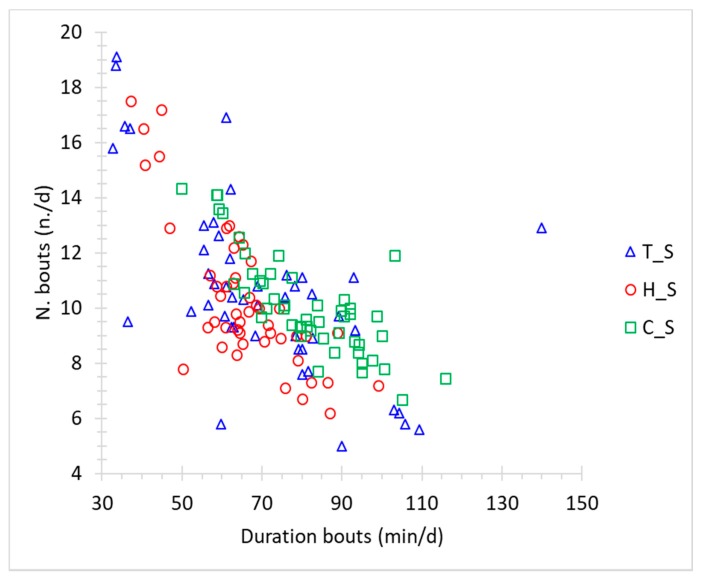
Relation between the daily number of lying bouts and their average duration in the three measurement periods (thermoneutral = T_S, hot = H_S and cold = C_S).

Instead, as previously mentioned, no clear relations were found between THI and number of lying bouts per day and between THI and the duration of bouts. However, Figure 8 reports a graph in which the average duration of lying bouts per day is related with the number of lying bouts, showing an inverse proportionality between the two variables. From the figure, it emerges that the dots referring to the cold period (C_S) are shown mostly in the lower-right part of the graph; hence, fewer lying bouts than in T_S and H_S are present, but with longer duration. The opposite condition occurs for the hot period, which is characterized by a higher number of lying bouts of low duration. 

## 4. Discussion

The results of this study show the differences in environmental conditions in the barn and its surroundings and in the behavioral response of dairy cows in eight farms located in the Lombardy region in Northern Italy. The relation between environmental conditions and behavioral response of dairy cattle was also investigated. 

From the results of this study, it emerges that although the geographical area in which the farms are included is the same, the specific farm characteristics and micro-climate affect the behavior of the reared cows. Moreover, the results make clear that supporting farmers with monitoring instrumentation is effectively useful because it allows us to analyze the variables that affect the livestock farming system and animal health and welfare. Therefore, PLF is a valid system to support farmers with decisions that regard animals and the barn management, which has positive effects on the productivity, health and efficiency of the dairy cattle [32,33,34]. 

Indeed, all results obtained from the monitoring surveys and data processing show that four of the analyzed farms performed better in respect to the others in response to heat stress events, probably because of the adequate barn management, insulation and cooling system. In fact, the farms C, D, E and F had better responses to the hot environmental conditions occurring during H_S, and also the respiration rate of the sampled cows was more constant during the three surveys. About the behavior, Farm D, in particular, showed almost constant cattle lying time during the year. This, together with the THI value maintained at lower than 70 along the year, can us bring to conclude that this farm is equipped with a well-insulated barn and/or an adequate cooling system for the rearing of dairy cows. Contrarily, the other four farms (i.e., A, B, G and H) highlight not negligible side effects during H_S; here, animals respond to the heat stress that occurs in H_S by increasing the standing time with respect to the lying one by reducing the duration of lying bouts and by increasing the respiration rate, even reaching panting conditions. For example, Farm B shows a high THI and a wide difference in the internal THI with respect to the external one during summer; moreover, it shows the lowest lying time during H_S and a high respiration rate. Both of these aspects bring us to conclude that there can be a problem in the barn construction that has results that are detrimental for animal welfare, production and reproduction aspects. The opposite condition occurred during C_S, when the cattle lying time in all farms was on average a good result considering the findings in the literature [25,35]. In particular, the THI was low; therefore, it did not negatively affect the behavior of the cows. 

In the cases in which the THI in the barn exceeded the one outside, the environmental conditions in the barn were insufficient in guaranteeing the animal welfare, and structural improvements should be planned. Among them, the forced ventilation is the solution most commonly adaptable to specific contexts and could represent one intervention for ameliorating animals’ conditions. Another option is the improvement of the roof insulation of barns, which is also an important aspect that can help to relevantly improve the harsh environment inside the barn. Moreover, as proposed by Schütz (2009) [11], the introduction of shades or the reduction of paved areas exposed to sun (if shades are already present, as in the surveyed farms) can also be a solution to poor design concerns. In Schütz (2009), [11] independent of the coat color and from the weather conditions, when either high solar radiation and/or high air temperature were present, cows preferred shaded areas protecting them from solar radiation. These authors also had similar findings to this study, as they got a higher mean body temperature when air temperature, THI and the heat load index increased. These variables had, as side effects, the changes in behavioral and physiological aspects that were attributed to the heat load. Other similar results were obtained by Brown-Brandl et al. (2005) [1], who found a relation between THI and the behavior of cows, THI and the respiration rate and the preference of cows to be in shaded areas with hot temperatures and their reduced ingestion. Similarly, Provolo and Riva (2008) [28] also found that the cow behavior was deeply affected by the THI, showing that even at lower values than the threshold of 72, important behavior changes emerged in the studied herds. Damasceno et al. (2019) [36] studied the temperature and illuminance conditions in the different parts of the barn by applying geostatistics, and it resulted in the fact that big variability can be present within barns depending on the distance from fans and from shades and showed that geostatistics can be useful to evaluate the microclimatic barn conditions. With regard to the lying time, Tullo et al. (2019) [35] found a relation among lying time and environmental conditions with the goal of having early warnings for unexpected behaviors. Although further studies are needed on this, the early warning could represent a key aspect in supporting farmers with herd management. The use of adoptable technology following PLF principles is indeed one of the most investigated aspects for current and near future livestock farming. PLF is a solution that involves an initial economic investment but that has undeniable positive effects on the herd management and on guaranteeing environmental, economic and social sustainable livestock productions [24,32,37,38,39]. Hence, its spread is increasing as it responds to the main requirements of new generations of farmers, consumers and policy makers that look for compromises for having efficient animal productions with satisfactory animal welfare, reduced environmental impact [40,41,42], low inputs purchase/expenses and low wear of resources [5,43]. 

An interesting aspect that could be object of a future research is to use the technology available from PLF to continuously monitor the behavioral responses of cows with respect to the environmental conditions, and alert the farmer at the occurrence of sub-optimal conditions in the barn that need his intervention (i.e., turning on the forced ventilation, introducing removable shades, etc.). 

## 5. Conclusions

Dealing with heat stress is becoming more and more important under the climate crisis conditions that affect the living situation of humans and animals. It has been demonstrated from other studies that heat stress affects the productive and reproductive performances of dairy cattle. In this study, the relation between environmental aspects and behavioral response of dairy cows was studied during a survey carried out in eight dairy barns located in Northern Italy. The possibility of measuring and monitoring the specific barn environmental conditions and the animal behavior has shown to be useful in evaluating the living conditions of dairy cattle and identifying the possible interventions to satisfy animal welfare. This survey helped highlight that wide differences can emerge even in a reduced geographic area and that it is important to have monitoring systems to control the environmental and behavioral trend of reared cows in every farm. In addition, the relation between THI and environmental and behavioral characteristics has shown strong results; therefore, if sufficient environmental conditions are not guaranteed already, intervening on the barn design (mainly cooling system and building insulation) can be a mitigation strategy to cope with heat stressing conditions.

## Figures and Tables

**Table 1:** Main characteristics of the monitored farms.

Variable	Unit	Farm A	Farm B	Farm C	Farm D	Farm E	Farm F	Farm G	Farm H
Monitored cows	n.	68	106	70	141	35	144	54	87
Milk production	kg/d	30.9	32.0	32.0	41.0	29.0	32.0	32.0	35.5
Bedding material	-	Straw	Straw	Mattress	Mattress	Mattress	Straw	Sand	Mattress
Ridge height	m	7.0	12.2	7.7	6.5	7.0	13.4	5.4	7.5
Roof slope	%	23	28	15	11	13	33	13	10
Upper opening		insufficient	adequate	adequate	insufficient	insufficient	adequate	adequate	insufficient
Barn orientation	-	E-W	NW-SE	NE-SW	E-W	NW-SE	NW-SE	NW-SE	E-W
Cooling system in the feeding area		fans + fogging	fans + fogging	fans + sprinkler	none	fans + sprinkler	none	fans + sprinkler	fans + sprinkler
Cooling system in the resting area	-	fans	none	fans	destratifiers	destratifiers	destratifiers	destratifiers	fans

**Table 2:** Mean, standard deviation and minimum and maximum values of the eight farms for the environmental results of temperature (T; °C), relative humidity (RH; %) and temperature-humidity index (THI), both in the barn and from a meteorological station close to the farm. The “T_S” refers to thermoneutral climate, “H_S” to hot climate and “C_S” to cold climate.

Period	Statistical Parameter	In the Barn			Meteorological Station		
		T (°C)	RH (%)	THI	T (°C)	RH (%)	THI
T_S	Mean (SD)	18.98 (1.95)	66.64 (9.39)	63.00 (2.15)	17.78 (2.11)	77.06 (14.24)	60.94 (1.69)
	Min-Max	14.59–21.50	56.80–79.85	58.35–67.03	13.39–20.90	55.93–100.00	57.42–63.47
H_S	Mean (SD)	26.30 (1.88)	69.31 (5.88)	71.84 (5.24)	25.90 (2.06)	69.97 (8.31)	67.55 (1.65)
	Min-Max	23.25–30.61	56.80–79.85	65.42–79.99	22.03–31.04	50.50–84.45	64.44–71.67
C_S	Mean (SD)	6.35 (1.99)	72.95 (6.80)	48.45 (3.82)	4.21 (2.07)	84.02 (10.85)	49.85 (1.70)
	Min-Max	2.56–10.14	54.50–84.88	40.46–53.37	−0.31–8.31	50.05–99.75	46.15–53.20

**Table 3:** Mean, standard deviation and minimum and maximum values of the eight farms for the behavioral results of lying and standing time (h/d), and of the number (n./d) and duration (min/bout) of lying bouts. The “T_S” refers to thermoneutral climate, “H_S” to hot climate and “C_S” to cold climate.

Survey	Statistical Parameters	Behavior			
		Standing (h/d)	Lying (h/d)	N. Bouts (n./d)	Duration Bouts (min)
T_S	Mean (SD)	13.4 (1.52)	10.60 (1.52)	10.70 (3.30)	69.97 (22.13)
	Min-Max	9.95–15.50	8.50–14.05	5.0–19.10	32.63–139.98
H_S	Mean (SD)	13.96 (1.40)	10.04 (1.40)	10.36 (2.66)	65.44 (13.05)
	Min-Max	11.34–17.54	6.46–12.66	6.20–17.50	37.20–99.14
C_S	Mean (SD)	11.94 (1.09)	12.06 (1.09)	10.06 (1.79)	81.42 (14.23)
	Min-Max	9.61–14.77	9.24–14.39	6.67–14.33	49.80–115.84

**Table 4:** Lying time (h/d) per farm during H_S.

Farm	Lying Time (h/d)
A	9.56bc
B	7.98d
C	10.49b
D	12.42a
E	9.00cd
F	10.67b
G	9.94bc
H	10.26b
